# The effect of tele-nursing based motivational interviewing on self-efficacy, self-management and metabolic control parameters in individuals with type 2 diabetes: randomized controlled study

**DOI:** 10.1007/s11845-025-03916-5

**Published:** 2025-02-21

**Authors:** Eda Kılınç İşleyen, İrem Nur Özdemir, Şengül Aydın Yoldemır

**Affiliations:** 1 Faculty of Health Sciences, Public Health Nursing Department, Uşak University, Uşak, Turkey; 2 Hamidiye Faculty of Nursing. Public Health Nursing Deparment, Health Sciences University, Istanbul, Turkey; 3 Kanuni Sultan Süleyman Training and Research Hospital, Istanbul, Turkey

**Keywords:** Motivational interview, Self-efficacy, Self-management, Tele-nursing, Type 2 diabetes

## Abstract

**Aim:**

The study was carried out to investigate the effect of tele-nursing based motivational interviewing on diabetes self-efficacy, self-management, and metabolic control parameters in individuals with Type 2 diabetes.

**Method:**

A parallel-group randomized controlled trial. The study was completed with 70 participants (intervention: 36; control: 34). The data were collected using the Socio-demographic and Health Related Questionnaire, the Diabetes Self-Efficacy Scale, the Diabetes Self-Management Scale. The intervention group received eight sessions tele-nursing based motivational interviewing. Instruments were administered to both groups before the intervention, at the end of the last motivational interviewing session (post-test, 3rd-month), and at 6th-month follow-up. The data were analyzed using chi-square test, independent sample t-test, and one-way ANOVA.

**Results:**

In the pre-test, there was no difference between the intervention and control groups in terms of independent variables (*p* > 0.05). Self-efficacy and self-management scores increased in the post-test and follow-up test of the group to which telenursing-based MI was applied, and there was a difference between the groups (*p* < 0.05). FBG and triglyceride levels of the intervention group decreased significantly in the post-test and follow-up test (FBG = 217.46 ± 73.88, 166.13 ± 50.71, and 161.41 ± 50.50, respectively; triglyceride = 225.28 ± 148.32, 159.68 ± 68.62, and 161.09 ± 73.06, respectively) (*p* < 0.05). HbA1c% level decreased significantly only in the post-test. However, no significant differences were found in terms of other metabolic parameters (*p* > 0.05).

**Conclusions:**

This result shows the positive effectiveness of tele-nursing based MI intervention on self-efficacy, self-management, HbA1c%, FBG, triglyceride. Public health nurses should be provided with tele-nursing based MI to individuals with type 2 diabetes in primary health care institutions.

Study registration.

The study was registered in ClinicalTrials NCT05628259 (prospective).

Trial registration number and date of registration for prospectively registered trials.

The study was registered in ClinicalTrials NCT05628259 (prospective). 2023–02-01.

## Introduction

Diabetes mellitus (DM) is a metabolic disorder that affects the body’s ability to regulate blood glucose levels. The prevalence of diabetes worldwide is a serious public health problem [1]. Type 2 diabetes mellitus (T2DM) develops as a result of the inability to use the insulin hormone effectively or to produce it insufficiently. This causes blood sugar levels to remain chronically high and causes long-term health problems. According to the International Diabetes Federation (IDF), it is estimated that approximately 537 million adults in the world have diabetes in 2021, and this number is predicted to reach 783 million by 2045 [2]. It shows that there are approximately 7 million diabetic patients between the ages of 20–79 in Türkiye and this figure corresponds to approximately 15% of the total adult population [2]. It is emphasized that diabetes is an increasing public health problem in Türkiye and the need to adopt prevention, early diagnosis, and effective management strategies [3, 4].

Individuals with T2DM are at high risk for cardiovascular mortality and microvascular complications [5, 6]. In addition to glycemic stability, body mass index and lipid targets are also important in reducing health risks from diabetes [7]. Targets for (glycosylated hemoglobin: HbA1c%, high density lipoprotein: HDL, low-density lipoprotein: LDL, cholesterol, and Body Mass Index: BMI are not being met, although education and support as well as medications are available to treat each of these conditions (glycemic goal of < 7.0% HbA1c, BMI target numbers: 18.5 to 24.9, LDL cholesterol < 70 mg/dL). The goals of the ADA include optimizing glycemic control with intensive lifestyle therapy in individuals with type 2 diabetes, keeping triglyceride levels below 150, and keeping LDL cholesterol below 40 for men and below 50 for women [8].

Ensuring self-management of diabetes will contribute to the prevention of diabetes-related complications as well as protecting and improving health. Diabetes self-management can be influenced by many factors, including motivational factors and barriers, attitudes, beliefs, and level of knowledge [9]. Behavioral interventions to improve self-management of diabetes have shown improvements in diabetes control by targeting eating habits, physical activity, and medication taking [10]. Therefore, interventions using behavioral strategies may support and help improve the diabetes management of individuals with diabetes. One of these behavioral strategies is motivational interviewing (MI).

MI interventions have been investigated in a variety of target behaviors, resulting in extensive applications related to disease management behaviors and in healthcare settings [11]. MI emerged to help people uncover their motivation for change. MI is an evidence-based approach intentionally delivered by someone trained in the practice to help make it easier for individuals to make decisions for themselves about making lifestyle and behavior changes [11]. MI is a clinical method that requires skill and reveals the internal motivation of individual with type 2 diabetes to choose positive health behaviors. The essential components of the MI spirit have been described as cooperation, acceptance, and support of patient autonomy [12]. There are four processes of MI: “engage,” “focus,” “elicit,” and “plan” [12]. MI practice involves the flexible and strategic use of some basic communication techniques, particularly those shared with other forms of person-centered counseling. These techniques are essential throughout the four processes of MI described above. These techniques facilitate rapid participation by giving the patient an active role during the interview. These are O: “Open Questions,” A: “Affirmation,” R: “Reflections,” and S: “Summaries.” [12, 13] Although MI has demonstrated benefits in improving outcomes for multiple diseases, its potential effectiveness in influencing outcomes in diverse adult populations with type 2 diabetes is particularly noteworthy [14]. Studies conducted in recent years have determined that MI has a positive effect on diabetes self-efficacy and self-management [15, 16]. Self-efficacy in diabetes refers to individuals’ beliefs and abilities to make decisions regarding diabetes management and to exhibit the necessary behaviors in this process. Self-efficacy reflects individuals’ sense of competence regarding the self-care skills required for diabetes control [17]. As the level of diabetes self-efficacy increases, diabetes self-management increases. Diabetes self-management includes medical nutrition therapy, exercise, medical treatment, regular blood glucose monitoring, and stress management [9, 17]. After the completion of the motivational interview process in individuals with type 2 diabetes, it was ensured that they regularly monitored their blood glucose, increased their exercise and fruit and vegetable intake, used their medications regularly, reduced alcohol and cigarette use, decreased hospitalizations, and improved glycemic control [11, 17].

With developing technology, the nurse can monitor the patient, provide consultancy, education, and nursing care by using remote telecommunication technologies. Recently, it has been observed that MI intervention for individuals with diabetes is carried out over the phone [18–21]. When telephone-based motivational interviewing interventions for individuals with diabetes are examined, promising improvements are observed in diabetes self-efficacy, self-management, HbA1c%, lipid and physical activity behavior [18, 20–22]. In addition to one-on-one or group interactions, the ADA recommends delivering lifestyle interventions via telehealth, such as incorporating person-centered communication into diabetes self-management education and support [23]. The study was carried out to investigate the effect of tele-nursing based motivational interviewing on diabetes self-efficacy, self-management, and metabolic control parameters in individuals with Type 2 diabetes.

**Hypotheses**

- **H1–1:** There is a significant difference between the intervention and control groups regarding diabetes self-efficacy.
- **H1-2:** There is a significant difference between the intervention and control groups diabetes self-management.
- **H1-3:** There is a significant difference between the intervention and control groups metabolic control parameters.

## Material and method

### Study design

This study is a single-blind randomized controlled parallel-group study trial. In the study, pre-test, post-test, and follow-up test were applied.

### Participants, sample size and power

This study was conducted with adults with Type 2 diabetes who were registered to the Diabetes Outpatient Clinic of a Training and Research hospital located in Istanbul/Türkiye between February 2023 and October 2023. G*Power 3.1.9.2. program was used to calculate the sample size. In the research, the sample size was calculated with reference to a similar study on the subject [24]. In the reference study, the effect size was calculated as 0.65 based on the post-intervention BMI variable score averages and standard deviation values of the individuals in the intervention and control groups. According to this effect size, in the analysis made by considering 5% margin of error (α) and 80% (1-β) power, it was determined that a total of 60 individuals with type 2 diabetes, including at least 30 intervention and 30 control groups, should constitute the sample of the study. The inclusion criteria of the study were as follows: 1) being over 18 years old, 2) receiving insulin or insulin + oral therapy, 3) diagnosis of type 2 diabetes at least one year ago, 4) having a BMI of 25 and above, 5) HbA1c% value of 7 and above, 6) access to phone, 7) voluntarily agreeing to participate in the study. The exclusion criteria of the study were as follows: 1) the individual has a physical disability caused by an extremity that prevents physical activity behavior have a physical disability. In this study, 220 patients were evaluated in terms of compliance with the criteria, and 71 patients who met the criteria were included in randomization.

### Randomization and blinding

**Fig. 1:**
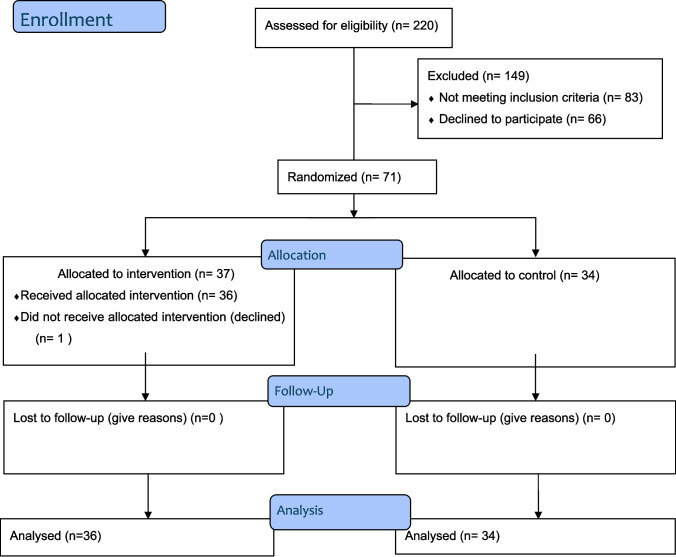
CONSORT diagram

Patients who met the criteria were identified in the outpatient clinic by the specialist physician who was the third researcher of the article. These patients, identified during the day, were divided into intervention and control groups by a simple random sampling method (envelope method) by an independent researcher. Initially, 37 intervention and 34 control groups were created. One person from the intervention group was excluded from the beginning of the study because he did not participate in the study at all. Intention to Treat (ITT) Per Protocol analyses were performed in the study to maintain randomization balance and prevent bias. This study resulted in 36 intervention and 34 control groups. The sample selection process, carried out according to CONSORT criteria, is shown in Fig. 1. Additionally, a single blinding method (participant) was used in this study.

### Variables of the research

The dependent variables of this study are diabetes self-efficacy, diabetes self-management and metabolic control parameters. The independent variable of this study tele-nursing based motivational interviewing.

### Outcome measures

In data collection the socio-demographic and health-related questionnaire, diabetes self-efficacy scale, diabetes self-management scale and metabolic control parameters were used.

#### Socio-demographic and health related questionnaire

This questionnaire was created by the researcher, using the literature on the subject to obtain the socio-demographic and health-related characteristics of the individuals included in the study [19, 25–27]. In this questionnaire, there are 22 questions in total, such as age, gender, education, duration of diabetes, treatment type, smoking, drinking, and metabolic parameters.

#### Diabetes self-efficacy scale (DSES)

It was developed by western culture by Van Der Bijl et al. (1999) to determine the perception of individuals diagnosed with type 2 diabetes about their own power in performing their own care activities in diabetes management. It was adapted into Turkish by Kara et al. (2006). The scale consists of three sub-dimensions as a result of the Turkish adaptation study [28]. These subdimensions are diet, exercise, and medical treatment. The lowest score on the scale is 20 and the highest score is 100. According to the overall average score obtained in the evaluation of the scale, individuals are said to have low/medium/high levels of self-efficacy, and as the score increases, their self-efficacy is high. The Cronbach alpha value of the scale is 0.892. The Cronbach alpha value for our study was 0.915.

#### Diabetes self-management scale (DSMS)

This scale was developed by Schmitt et al. (2013) to examine the relationship of diabetic patients with diabetes self-management and glycemic control. The validity and reliability study of the Turkish Diabetes Self-Management Questionnaire was conducted by Eroğlu and Sabuncu (2019) [29]. The scale consists of 16 items and 4 sub-scale: Glucose Management, Diet Control, Physical Activity and Health Services. The Cronbach’s alpha value of the scale is 0.85. A minimum of 0 and a maximum of 10 points are obtained from the scale. If an item is skipped, it is evaluated as −3 points. As the score gets closer to 10, diabetes self-management increases. The Cronbach alpha value for our study was 0.867.

### Metabolic control parameters

These parameters include BMI, waist circumference, FBG, HbA1c%, LDL, HDL, total cholesterol, and triglyceride. The FBG, HbA1c%, LDL, HDL, total cholesterol and triglyceride values ​​of the patients were examined in this laboratory by contacting the physician. BMI values ​​of all patients were measured by the researcher in the training room of the hospital. A wall-mounted height meter fixed to the wall was used for height measurement. In the measurements, the shoes of the individuals were removed, the head and body were positioned upright, the soles of the feet flat on the ground. The BMI value was calculated by dividing the individual’s body weight (kg) by the square of the height (m^2^) (BMI = kg/m^2^). BMI values of individuals were evaluated according to the BMI classification table created by WHO [30].

### Intervention

EKİ has a "Motivational Interviewing Techniques Training" certificate. She applied Motivational Interviewing Techniques. The MI program based on the telenursing approach was prepared by the researchers based on the literature for the individuals in the intervention group [18–21, 26, 31, 32]. In this study, the MI procedure includes four sessions of motivational interviewing by phone for each individual in the first month of the intervention, and four sessions of MI by phone in the next two months of the intervention, for a total of eight interviews. Pre-tests were collected for the first month, and then MI intervention was applied for three months. This intervention was applied to the intervention group in addition to the routine care provided by the hospital. MI intervention took approximately 45–60 min via phone call (Table 1.).

**Table 1:** Interventions

Activity	Description	
Pre-test (month 1)	Demographic and health information questionnaire, diabetes self-efficacy scale, diabetes self-management scale, BMI, waist circumference, FBG, HbA1c (%), LDL, HDL, total cholesterol, triglyceride	
MI intervention session 1 (month 2)	• Investigating the patient’s behavioral change stage • Asking the patient to detail the impact of diabetes on their life and share the benefits of diabetes self-management • Exploring goals and values, offering topic bubbles to set personalized goals • OARS techniques (open-ended questions. affirming. reflective listening and summarizing) were used and common goals were determined • Investigating the patient’s motivation and readiness for change using readiness • Summarizing the entire process and presenting it to the individual	
MI intervention session 2, 3, 4 (month 2)	• Exploring the patient’s stage of behavior change and progress with the self-management plan • For the individual to maintain the change in behavior the researcher receives information about the change in the individual’s health and support the positive changes in her / his life • Investigating barriers and solutions to behavior change • Investigating whether the patient is ready for change using a readiness chart	
MI intervention session 5, 6, 7 (month 3, 4)	• Investigating barriers and solutions to behavior change • Supporting the individual’s behavioral change and providing motivation • If the individual exhibits positive behavior in line with his/her goals, appreciate the individual and continue cooperation	
MI intervention session 8 (month 4)	• The researcher summarizes the entire process, appreciating the individual’s collaborative effort towards change throughout the process. For example, “Congratulations on your three-month change effort. You’ve come a long way. The change here was all about your own motivation and desire. I hope you will continue these behavioral changes in the future.” • The researcher receives feedback on what the individual thinks throughout the entire interview and ends the Motivational Interviewing process	
Post-test (month 5)	• Diabetes self-efficacy scale, diabetes self-management scale, BMI, waist circumference, FBG, HbA1c (%), LDL, HDL, total cholesterol, triglyceride	
Follow-up test (month 8–9)	• Diabetes self-efficacy scale, diabetes self-management scale, BMI, waist circumference, FBG, HbA1c (%), LDL, HDL, total cholesterol, triglyceride	

MI: Motivational interview, BMI: Body mass index, FBG: fasting blood glucose, HbA1c: Hemoglobin A1C, LDL: Low-density lipoprotein, HDL: High-density lipoprotein

### Data collection procedures

The data collection process of the study was completed between February 2023 and October 2023. Before starting the intervention, pre-tests were applied to the individuals in both groups and the necessary measurements (BMI, waist circumference, FBG, HbA1c%, LDL, HDL, total cholesterol, and triglyceride) were measured. The scales are filled with self-report. The data collection time for each of the pre-test, post-test and follow-test took an average of 30–40 min. After the pre-test, motivational interview was applied to the intervention group. The control group received only routine care provided by nurses in the hospital.

### Data analysis

The SPSS 22 program was used for data analysis. Descriptive statistics were used in the analysis of socio-demographic and health-related data of the intervention and control groups. Homogeneity of the groups was evaluated with Pearson chi-square and independent sample t-test. Skewness and kurtosis values were used to fit the normal distribution. If the skewness and kurtosis values are between −2 and + 2, they show a normal distribution [33]. This value was taken as a reference in our study. Independent sample t-test was used to compare the mean scores between groups in pre-test, post-test, and follow-up test measurements. One-way Anova was used in repeated measurements to compare the changes in the mean scores between the follow-ups within the groups. The partial η2 value was used to calculate the effect size. This value is accepted as small if it is 0.01, while the value was considered as moderate if it is 0.06 and large if it is 0.14 [34]. The statistical significance level in the study was *p* < 0.05.

*Ethical considerations.*

Ethics committee approval of the study was received from Pamukkale University Non-Interventional Clinical Research Ethics Committee (Date: 05.12.2022, Number: 2022–23-13). Official permission was obtained from Bakırköy Dr. Sadi Konuk Education and Training Hospital for the diabetes polyclinic where the study was conducted (Date: 16.01.2023, Number: E-14679818–771-207,519,667). In addition, written consent was obtained from all individuals in the intervention and control groups included in the study.

## Results

The mean age of the intervention group was 55.22 ± 7.07, while that of the control group was 55.55 ± 8.31 (*p* > 0.05). The mean age of the intervention group was 13.58 ± 8.47, while that of the control group was 12.85 ± 9.64 (*p* > 0.05). The averages and percentages of baseline characteristics and metabolic control parameters (BMI, waist circumference, FBG, HbA1c%, LDL, HDL, total cholesterol, and triglyceride) of the intervention and control groups are given in Table 2. As a result of the analysis, it was determined that there was no difference between the individuals in the intervention and control groups in terms of baseline characteristics and metabolic control parameters, and both groups were homogeneously distributed (*p* > 0.05) (Table 2).

**Table 2:** Baseline characteristics in the intervention and the control group

Characteristics	Intervention group (*n* = 36)		Control group (*n* = 34)		Statistical analysis
Age (X ± SD)	55.22 ± 7.07		55.55 ± 8.31		t = −0.183 *p* = 0.855
Diabetes duration (year)	13.58 ± 8.47		12.85 ± 9.64		t = 0.337 *p* = 0.737
Years of insulin use	7.94 ± 6.49		8.79 ± 10.05		t = −0.422 *p* = 0.674
BMI	32.454.86		30.81 ± 5.94		t = 1.261 *p* = 0.212
Waist circumference	104.30 ± 9.15		100.64 ± 11.58		t = 1.470 *p* = 0.146
FBG	217.46 ± 73.88		215.00 ± 104.80		t = 0.114 *p* = 0.909
HbA1c (%)	9.53 ± 1.87		9.31 ± 2.42		t = 0.434 *p* = 0.666
LDL	114.00 ± 41.12		114.54 ± 43.16		t = −0.054 *p* = 0.957
HDL	45.31 ± 11.95		55.50 ± 41.47		t = −1.414 *p* = 0.162
Total cholesterol	197.83 ± 48.39		201.55 ± 54.88		t = −0.302 *p* = 0.764
Triglyceride	225.28 ± 148.32		193.05 ± 113.15		t = 1.018 *p* = 0.313
	**n**	**%**	**n**	**%**	
Gender					
Female	22	61.10	19	55.90	X^2^ = 0.197 *p* = 0.657
Male	14	38.90	15	44.10	
Education status					
Primary school	28	77.80	29	85.30	X^2^ = 1.895 *p* = 0.388
High School	7	19.40	3	8.80	
University or above	1	2.80	2	5.90	
Marital Status					
Married	34	94.40	31	91.20	X^2^ = 0.282 *p* = 0.596
Single	2	5.60	3	8.80	
Working					
No	24	66.70	28	82.40	X^2^ = 2.252 *p* = 0.133
Yes	12	33.30	6	17.60	
Income					
Income is less than expenses	16	44.10	18	52.90	
Income equals expenses	20	55.60	13	38.20	X^2^ = 4.549 *p* = 0.103
Income exceeds expenses	0	0.00	3	8.80	
Smoking					
I have never smoked	23	63.90	19	55.90	X^2^ = 0.524 *p* = 0.769
I quit	4	11.10	4	11.80	
Yes, I smoke	9	25.00	11	32.40	
Drinking					
I have never drunk	35	97.20	30	88.20	X^2^ = 2.329 *p* = 0.312
I quit	0	0.00	1	2.90	
Yes, I am drinking	1	2.80	3	8.80	
Treatment type					
Insulin	11	30.60	6	17.60	X^2^ = 1.585 *p* = 0.208
Insulin + oral	25	69.40	28	82.40	
Diabetes-related complication					
No	13	36.10	16	47.10	X^2^ = 0.864 *p* = 0.353
Yes	23	63.90	18	52.90	
Presence of other chronic diseases					
No	8	22.20	13	38.20	X^2^ = 2.135 *p* = 0.144
Yes	28	77.80	21	61.80	
Is there any other medication used regularly?					
No	9	25.00	11	32.40	X^2^ = 0.463 *p* = 0.496
Yes	27	75.00	23	67.60	

Abbreviations: *X* Mean; *SD* Standard deviation; *t* t test; *X*^*2*^ Pearson Chi-square test

There was no difference in diabetes self-efficacy (intervention group: 66.36 ± 7.75, control group: 65.38 ± 13.43) and self-management (intervention group: 25.44 ± 6.50, control group: 23.32 ± 7.27) between the intervention and control groups before the intervention (*p* > 0.05). A significant difference in diabetes self-efficacy was found between the groups in the post-test (intervention group: 84.41 ± 12.74, control group: 70.11 ± 10.83) and follow-up test (intervention group: 84.22 ± 13.16, control group: 70.02 ± 10.95) (*p* < 0.05). A significant difference in diabetes self-management was found between the groups in the post-test (intervention group: 32.75 ± 5.57, control group: 26.11 ± 9.00) and follow-up test (intervention group: 32.25 ± 6.26, control group: 26.17 ± 8.98) (*p* < 0.05). In the post-test and follow-up test, the diabetes self-efficacy and self-management scores of the intervention group increased, while the control group remained similar. It was found that the MI intervention applied to the intervention group had a high effect on diabetes self-efficacy (η2 = 0.622) and self-management (η2 = 0.622). This result shows the effectiveness of tele-nursing based MI intervention on self-efficacy and self-management (Table 3).

**Table 3:** Comparison of the diabetes self-efficacy, the diabetes self-management and metabolic control parameters mean scores of groups within and between groups

Time	Pre-test X ± SD	Post-test X ± SD	Follow-up test X ± SD	Within-group difference	η^2^
Group					
Diabetes self-efficacy total score					
Intervention	66.36 ± 7.75	84.41 ± 12.74	84.22 ± 13.16	F = 27.945. *p* = 0.000*	0.622
Control	65.38 ± 13.43	70.11 ± 10.83	70.02 ± 10.95	F = 2.692 *p* = 0.083	0.144
Difference between groups	t = 0.376 *p* = 0.708	t = 5.042 *p* = 0.000*	t = 4.882 *p* = 0.000*		
Diabetes self-management total score					
Intervention	25.44 ± 6.50	32.75 ± 5.57	32.25 ± 6.26	F = 31.145 *p* = 0.000*	0.647
Control	23.32 ± 7.27	26.11 ± 9.00	26.17 ± 8.98	F = 4.477 *p* = 0.019*	0.219
Difference between groups	t = 1.288 *p* = 0.202	t = 5.416 *p* = 0.000*	t = 4.921 *p* = 0.000*		
BMI					
Intervention	32.45 ± 4.86	30.42 ± 4.09	30.78 ± 4.40	F = 1.184 *p* = 0.168	0.100
Control	30.81 ± 5.94	30.43 ± 5.38	30.42 ± 5.38	F = 0.663 *p* = 0.522	0.040
Difference between groups	t = 1.261 *p* = 0.212	t = −0.009 *p* = 0.993	t = 0.309 *p* = 0.758		
Waist circumference					
Intervention	104.30 ± 9.15	101.11 ± 10.53	101.05 ± 9.71	F = 2.392 *p* = 0.107	0.123
Control	100.64 ± 11.58	98.91 ± 9.82	98.64 ± 9.24	F = 0.884 *p* = 0.423	0.052
Difference between groups	t = 1.470 *p* = 0.146	t = 0.902 *p* = 0.370	t = 1.061 *p* = 0.292		
FBG					
Intervention	217.46 ± 73.88	166.13 ± 50.71	161.41 ± 50.50	F = 10.418 *p* = 0.000*	0.380
Control	215.00 ± 104.80	212.27 ± 96.47	207.08 ± 99.23	F = 1.126 *p* = 0.337	0.066
Difference between groups	t = 0.114 *p* = 0.909	t = −2.525 *p* = 0.014*	t = −2.447 *p* = 0.017*		
HbA1c%					
Intervention	9.53 ± 1.87	7.77 ± 1.01	8.08 ± 1.01	F = 14.378 *p* = 0.000*	0.458
Control	9.31 ± 2.42	8.71 ± 2.25	8.66 ± 2.26	F = 3.166 *p* = 0.056	0.165
Difference between groups	t = 0.434 *p* = 0.666	t = −2.274 *p* = 0.026*	t = −1.381 *p* = 0.172		
LDL					
Intervention	114.00 ± 41.12	110.43 ± 37.88	113.83 ± 40.90	F = 0.319 *p* = 0.729	0.018
Control	114.54 ± 43.16	118.85 ± 35.23	118.16 ± 36.17	F = 0.590 *p* = 0.561	0.036
Difference between groups	t = −0.54 *p* = 0.957	t = −0.949 *p* = 0.346	t = −0.468 *p* = 0.641		
HDL					
Intervention	45.31 ± 11.95	48.83 ± 19.92	49.41 ± 20.83	F = 1.317 *p* = 0.281	0.072
Control	55.50 ± 41.47	50.07 ± 24.46	48.77 ± 23.95	F = 1.070 *p* = 0.355	0.063
Difference between groups	t = 0.032 *p* = 0.162	t = 0.732 *p* = 0.817	t = 0.886 *p* = 0.905		
Total cholesterol					
Intervention	197.83 ± 48.39	187.19 ± 50.34	197.75 ± 47.34	F = 0.980 *p* = 0.386	0.055
Control	201.55 ± 54.88	204.56 ± 46.41	202.20 ± 45.11	F = 0.487 *p* = 0.619	0.030
Difference between groups	t = 0.448 *p* = 0.764	t = −1.441 *p* = 0.154	t = −0.403 *p* = 0.688		
Triglyceride					
Intervention	225.28 ± 148.32	159.68 ± 68.62	161.09 ± 73.06	F = 3.847 *p* = 0.031*	0.185
Control	193.05 ± 113.15	209.79 ± 116.21	207.32 ± 115.80	F = 1.326 *p* = 0.280	0.077
Difference between groups	t = 1.018 *p* = 0.313	t = −2.212 *p* = 0.030*	t = −2.009 *p* = 0.048*		

Abbreviations: *X* Mean; *SD* Standard deviation; * Significant; *t* Independent sample t test; *F* One-way ANOVA with repeated measures; *T0* Pre-test; *T1* Post-test; *T2* Follow-up test

A significant difference was found in the FBG level in the pre-test and post-test in intervention group (217.46 ± 73.88 vs 166.13 ± 50.71) (*p* < 0.05). A significant difference was found between the groups in the HbA1c% level in the posttest (intervention group: 7.77 ± 1.01, control group: 8.71 ± 2.25) (*p* < 0.05). It was determined that the MI intervention applied to the intervention group had a high effect on FBG (η2 = 0.380) and HbA1c% (η2 = 0.458). A significant difference was found between the groups in triglyceride levels in the post-test (intervention group: 159.68 ± 68.62, control group: 209.79 ± 116.21) and follow-up test (intervention group: 161.09 ± 73.06, control group: 207.32 ± 115.80) (*p* < 0.05). The triglyceride value of the intervention group decreased significantly in the post-test and follow-up test (*p* < 0.05). It was determined that the MI intervention applied to the intervention group had a high effect on triglyceride (η^2^ = 0.185). However, no significant differences were found in terms of other metabolic parameters (*p* > 0.05).

## Discussion

Ensuring self-management and metabolic control in individuals with diabetes is of great importance to prevent complications [7, 8]. Tele-nursing based MI intervention for individuals with diabetes was applied for eight sessions in total. Our findings show that tele-nursing-assisted MI intervention for uncontrolled T2DM patients positively affects diabetes self-efficacy and self-management levels.

In this study, the significant increase in the diabetes self-efficacy score of the tele-nursing based MI intervention group can be associated with personal support and follow-up, accessibility, information, motivation and goal setting [18]. Tele-nursing provided patients with diabetes with continuous and personal support, thus patients felt more secure and were more motivated about diabetes management. In addition, since patients were informed about diabetes management during motivational interviews, it had a direct positive effect on increasing their self-efficacy [18, 21, 35]. Existing literature includes experimental studies highlighting the potential effectiveness of tele-nursing based MI intervention on diabetes self-efficacy and self-management [19, 32, 36, 37]. In the study conducted by Kılınç and Kartal, diabetes education and MI were applied to individuals with T2DM based on the Information, Motivation and Behavioral Skills Model. MI intervention was conducted via WhatsApp application in six sessions. As a result of the study, similarly, the self-efficacy score of the diabetic individuals in the intervention group increased after the intervention [32]. In Swoboda et al. (2017) study, individuals in the intervention group received seven sessions of telephone support intervention. In parallel with our study, the diabetes self-efficacy score of the intervention group increased significantly after the intervention [37].

In this study, the group that received tele-nursing based MI intervention had a significant increase in diabetes self-efficacy scores, as well as an increase in self-management scores. The reason for this situation is that individuals’ self-care behaviors regarding healthy nutrition, exercise, blood glucose monitoring, and medication use increased. The application of telephone-supported MI to diabetic individuals helped individuals set goals and be motivated to achieve these goals [18, 21, 35]. Similar to our study, the study by Sawaengsri et al. (2023), four sessions of short MI intervention were applied to individuals with type 2 diabetes over the phone. After the intervention, an increase in self-management and medication compliance were observed in individuals with type 2 diabetes [19]. In these studies, the increase in diabetes self-management after the intervention shows the significant effect of telephone MI intervention performed in a short time. Tele-nursing based MI intervention was found to increase people with diabetes’ motivation and/or health goals, thereby improving their self-management levels of dieting, physical activity, self-monitoring, medication taking, and foot care. Patients who improve DM self-management are also more likely to improve glycemic control [36].

In this study, the group that received tele-nursing based MI intervention had a significant decrease in FBG, HbA1c% and triglyceride levels. However, there was no significant difference between the intervention and control groups in terms of BMI, waist circumference, LDL, HDL, and total cholesterol. Although a decrease in FPG and HbA1c is observed over a 6-month period, it may not be seen in BMI, waist circumference, LDL, HDL values. In the study of Sawaengsri et al. (2023), four sessions of short MI intervention were applied to individuals with type 2 diabetes over the phone. After the intervention, an increase in self-management and medication compliance and a significant decrease in FBS were observed in individuals with type 2 diabetes. No significant decrease was observed in the HbA1c% value [19]. In these studies, the increase in diabetes self-management after the intervention shows the significant effect of telephone MI intervention performed in a short time. However, the reason why there is no significant difference in the HbA1c% value is that the last test was measured in a short period of only 8 weeks. Our results suggest that telenursing-assisted MI may be as effective as face-to-face MI sessions. Blackberry et al. (2007) study, a telephone coaching intervention based on eight sessions of motivational interviewing was implemented under the leadership of a nurse. In this study, there was no significant difference in HbA1c% levels between the intervention and control groups after the intervention. Browning et al. (2016) study, telephone and face-to-face MI intervention was applied to the intervention group, and as a result of the intervention, no difference was found between the groups in terms of HbA1c%, triglyceride, LDL and HDL. The results of cholesterol values in Browning et al. (2007) study is parallel to our study findings. It can be said that MI intervention needs to be followed up longer to have the expected effect on HbA1c% and triglyceride values over time. In Fischer et al. (2012) study, a telephone-based MI intervention was applied to individuals with diabetes with a 20-month follow-up. As a result of the study, the proportion of patients in the intervention group whose LDL level fell below 100 decreased significantly compared to the control group [35]. The fact that the LDL rate did not decrease in our study may be related to the implementation of a shorter intervention. Our results suggest that further experimental studies are needed to evaluate MI strategies incorporated into standard care to better understand the effectiveness of tele-nursing based MI intervention in diabetes management.

A meta-analysis by McDaniel et al. (2020) stated that MI-based telehealth applications showed promising results in individual with type 2 diabetes. For this reason, tele-nursing based MI is seen as a valuable approach that can be applied by nurses in primary health care institutions. Nurses, who play an important role in protecting and improving the health of individuals with type 2 diabetes, can take on the role of consultant and actively participate in interventions to ensure diabetes self-management and metabolic control. We recommend that future studies examine the effectiveness of telephone-based motivational interviewing intervention in individuals with diabetes over a longer period. Additionally, interaction can be made not only via phone call but also via tablet, computer or video-conference.

### Limitations

The significant improvement observed in patient outcomes should be interpreted with caution because individuals who agreed to participate in the study may be more likely to change or more prepared to change compared to the general population.

## Conclusion

In this study, a total of eight sessions of tele-nursing-based MI intervention were applied to individuals with type 2 diabetes. Self-efficacy, self-management and metabolic control parameters were evaluated in the study group before and after the intervention. After the intervention, a positive improvement was observed in the diabetes self-efficacy and diabetes self-management scores of the intervention group. Additionally, a decrease in FBG, HbA1c%, and triglyceride values was observed in the intervention group. However, no improvement was seen in BMI, waist circumference, LDL, HDL, and total cholesterol values. The results of this research are promising. For this reason, public health nurses should be provided with telephone based MI to individuals with type 2 diabetes in primary health care institutions and hospitals.

## Data Availability

The data supporting the findings of this study are available from the corresponding author upon reasonable request.
